# Collateral Impact of Mannose Supplementation on Metastatic Properties in Osteosarcoma Cell Models

**DOI:** 10.3390/biology15020127

**Published:** 2026-01-11

**Authors:** Ayami Morita, Toshifumi Hara

**Affiliations:** 1 Laboratory of Cellular Pharmacology, School of Pharmacy, Aichi Gakuin University, Nagoya 464-8650, Japan; ayami@dpc.agu.ac.jp; 2 Laboratory of Medicinal Biochemistry, School of Pharmacy, Aichi Gakuin University, Nagoya 464-8650, Japan; 3 Laboratory of Biochemistry, School of Pharmacy, Aichi Gakuin University, Nagoya 464-8650, Japan

**Keywords:** mannose, glycolysis, cancer progression, epithelial–mesenchymal transition

## Abstract

Mannose supplementation has emerged as a promising strategy to exploit the hidden vulnerabilities of cancer cells. However, the mannose-dependent effect on cellular functions remains unclear. Here, we investigated the collateral impact of mannose supplementation on the metastatic potential of osteosarcoma. Analysis of a paired osteosarcoma dataset showed significant upregulation of GPI in tumors compared with matched normal tissue, whereas other glycolysis- and mannose-metabolism genes showed non-significant upward trends. By using osteosarcoma cell lines, MG-63 carrying a pronounced epithelial-to-mesenchymal transition phenotype showed a dose-dependent inhibition of proliferation upon mannose supplementation. We also found that mannose-induced attenuation of cell growth was further enhanced, accompanied by marked reductions in migration and invasion ability. Consistently, qPCR analysis indicated that mannose supplementation downregulated key genes associated with metastasis. Collectively, our data reveal that mannose inhibits osteosarcoma not merely by dampening glycolysis but also by triggering, in part, MPI-linked mannose metabolism, which disrupts key drivers of motility-associated phenotypes. Together, these data show that pharmacologic mannose supplementation modulates cell growth- and motility-associated phenotypes in osteosarcoma cell models, accompanied by changes in epithelial-to-mesenchymal transition-associated transcripts.

## Simple Summary

Osteosarcoma is a rare but aggressive bone cancer that mainly affects children and teenagers, and its tendency to spread makes it very difficult to cure. Recently, supplementation with mannose, a natural sugar closely related to glucose, has been shown to slow the growth and spreading of cancer cells by exploiting their altered sugar metabolism, which has been explored as a metabolic perturbation in preclinical cancer models. This study examined osteosarcoma patient transcriptome data and found that GPI was significantly upregulated in tumor tissue compared with paired normal bone, whereas other glycolysis- and mannose-metabolism genes showed modest, non-significant upward trends. Then we investigated the effect of mannose supplementation in osteosarcoma cell lines and found the dose-dependent effect of mannose supplementation on growth retardation, especially in cells with high metastatic potential. Parallel with this finding, mannose supplementation also reduced their ability to migrate and to invade, as a progression marker of osteosarcoma. In addition, we showed that mannose supplementation repressed gene expression of several genes that drive metastasis. Our findings suggest mannose metabolism as a tractable vulnerability in osteosarcoma cell models and motivate future in vivo work to test whether modulating mannose availability can complement existing therapies.

## 1. Introduction

Osteosarcoma, the most prevalent primary bone tumor in children and adolescents, remains a clinical challenge due to its high-grade aggressiveness, early metastatic behavior, and poor prognosis [1]. Hallmark biological traits of genomic instability, heterogeneity, and metabolic adaptability suggest underlying vulnerabilities that remain largely unexploited in osteosarcoma despite multimodal treatment strategies against cancers [2]. One of the defining features of osteosarcoma is its marked metabolic reprogramming, most notably characterized by enhanced aerobic glycolysis, termed the Warburg effect, wherein cancer cells preferentially metabolize glucose through glycolysis even in the presence of oxygen [3,4,5].

Osteosarcoma displays a strong glucose dependency, reflected by high ^18^F-FDG uptake in clinical PET imaging [6,7]. Hypoxia and recurrent genomic alterations likely reinforce this glycolytic state under metabolic stress [8,9].

Beyond glycolysis, alternative monosaccharide pathways have emerged as potential metabolic vulnerabilities. Mannose is transported into cells and phosphorylated to mannose-6-phosphate, which can be routed into central carbon metabolism through mannose phosphate isomerase (MPI) [10]. In several cancer models, supraphysiologic mannose supplementation has been reported to suppress growth and to enhance chemotherapy responses, with sensitivity influenced by MPI expression [11,12].

Despite these advances, the relevance of mannose metabolism to osteosarcoma progression remains poorly defined [11,13]. First, whether glycolysis and mannose-metabolism genes show coordinated expression changes in osteosarcoma transcriptome resources remains unclear. Second, it is unknown whether mannose exposure modulates motility-associated phenotypes and EMT-associated transcriptional programs in osteosarcoma cells and to what extent MPI controls these responses.

Here, we hypothesized that mannose supplementation constrains motility-associated phenotypes in osteosarcoma cell models and that MPI expression modulates sensitivity to mannose. To address this, we assessed cell proliferation, transwell migration and invasion capacities, and EMT-associated transcripts, and we evaluated the impact of MPI in the context of osteosarcoma cell models.

## 2. Materials and Methods

### 2.1. Data Resource and Analysis

Transcriptome profiles from paired tumor and adjacent normal tissues of 18 osteosarcoma patients (GSE99671) were retrieved and processed for differential-expression analysis. Raw count data were utilized, and therefore upstream processing such as read quality control and alignment was performed by the original depositors. Prior to differential expression analysis, we assessed data integrity at the count level. We performed variance-stabilizing transformation (VST) and verified sample structure using Principal Component Analysis (PCA) and hierarchical clustering to ensure no outliers were present. Differential expression was analyzed with R software (version 4.5.1) using the DESeq2 package (version 1.40.2). Prognostic data of 88 osteosarcoma patients (GSE42352) were analyzed using the R2 Genomics Analysis Platform (https://hgserver1.amc.nl/; accessed on 27 June 2025). Overall survival curves were generated in the R2 platform using scan mode, which evaluates multiple expression cutoffs and selects the threshold that minimizes the log rank *p* value, with the platform also reporting a cutoff adjusted *p* value. Because this procedure is exploratory, we interpret these results as hypothesis generating.

### 2.2. Cell Cultures and Reagents

The human osteosarcoma cells U-2 OS and MG-63, obtained from the American Type Culture Collection (ATCC), were cultured in DMEM supplemented with 10% fetal calf serum (FCS) and 1% penicillin-streptomycin under 37 °C humidified 5% CO_2_ environment. Mannose supplementation was performed at the concentration specified for each experiment. Glucose and mannose were diluted in PBS and supplementation was performed at 25 mM. For the generation of *MPI*-knockdown MG-63 cells, we employed the Mission shRNA system (Sigma-Aldrich, St. Louis, MO, USA). We validated the five kinds of pre-designed shRNA for *MPI*, and only one shRNA (TRCN0000365267) was used in this study. The detail was described in Figure S2. The plasmid DNA for either non-targeting (shNT) or MPI (shMPI) was transfected into MG-63 cells, and then the cells were cultured in the presence of 2 μg/mL puromycin for 2 weeks to select stably shRNA-expressing cells.

### 2.3. RNA Isolation and Quantitative PCR (qPCR)

Total RNAs were isolated with the ISOGEN reagent (Nippon Gene, Tokyo, Japan), according to the manufacturer’s protocol. The quality and quantities of RNAs were determined with the nano-spectrometer DS-11 (DeNovix, Wilmington, DE, USA). 500 ng of RNA was subject to reverse-transcription with iScript super mix (Bio-Rad, Hercules, CA, USA), and then qPCR was performed with gene-specific primers and THUNDERBIRD^®^ Next SYBR™ qPCR Mix (TOYOBO, Tokyo, Japan) using the StepOnePlus Real-time PCR System (Thermo Fisher Scientific, Waltham, MA, USA). The relative expression level was calculated using the level of the reference gene *SDHA* by the ΔΔCq method. DNA sequences of the primers were described in Table S1.

### 2.4. Cell Proliferation Assay

Cells were seeded at 2 × 10^3^ cells in 96-well plates. Cell proliferation was determined at the indicated time point using a Cell Counting Kit-8 (CCK-8; Dojindo, Tokyo, Japan). After incubation of CCK-8 for two hours, absorbance at 450 nm was measured with the microplate reader, Spark (TECAN, Männedorf, Switzerland). For control, a well containing culture medium and CCK-8 without cells was used.

### 2.5. Cell Migration Assay and Invasion Assay

Cells were cultured in FCS-depleted conditions for 24 h, and then 2 × 10^5^ cells were placed on a transwell culture insert (Corning, Corning, NY, USA) in FCS-depleted conditions. A transwell culture insert was pre-coated with Matrigel (Corning, Corning, NY, USA) only for the invasion assay before the set of cells. A transwell culture insert was placed into a 24-well companion plate containing 10% FCS culture medium. After 48 h, the cells that did not migrate or invade through the membrane pore were removed with a cotton swab. Cells on the outer membrane were fixed and stained with crystal violet. Pictures of the membrane were taken, and the invaded cells were counted.

### 2.6. Statistical Analysis

All in vitro experiments were performed as independent biological replicates (n is stated in each figure legend). Continuous outcomes were analyzed using two-sided tests. We utilized parametric tests because data from quantitative PCR and cell viability assays in controlled biological replicates typically approximate a normal distribution. Prior to testing, we verified that the variance between groups was comparable. For two-group comparisons, we used *t*-test (unpaired) or paired *t*-test (paired design), as appropriate. For multi-group comparisons, one-way or two-way ANOVA was applied with prespecified multiple comparison procedures (Dunnett for comparisons versus control). For survival analysis, we used the R2: Genomics Analysis and Visualization Platform to generate Kaplan–Meier overall survival curves and to compare groups using a two-sided log rank (Mantel–Cox) test. The level of statistical significance was set at 5% and 1%, and the results are shown (* *p* < 0.05, ** *p* < 0.01).

## 3. Results

### 3.1. The Importance of Glycolytic and Mannose Metabolic Pathways in Osteosarcoma

**Figure 1:**
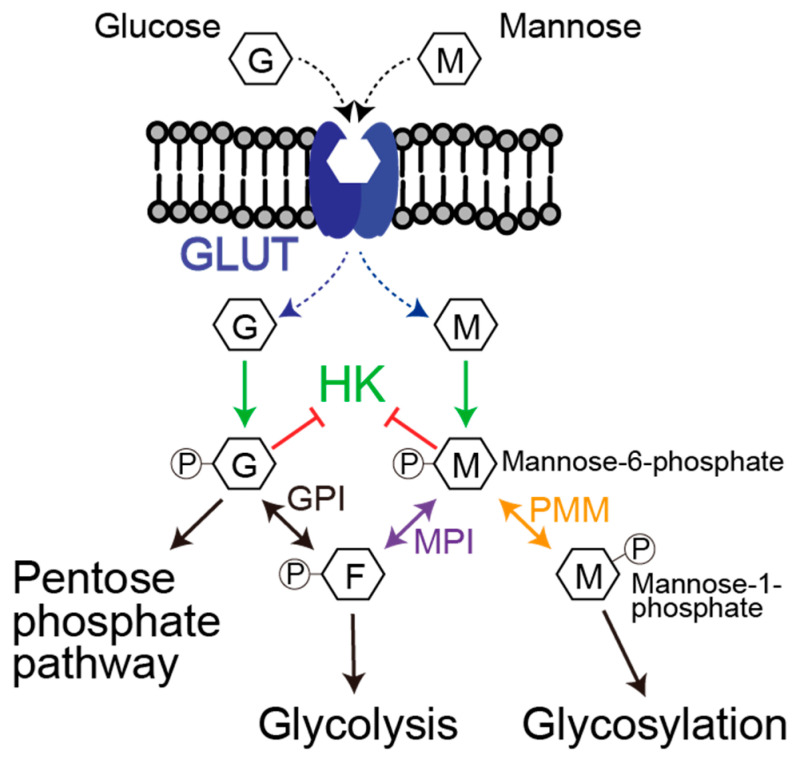
Pathways to intracellular metabolism of glucose and mannose. The schematic shows the intracellular metabolism of glucose and mannose. Glucose and mannose are taken up via identical GLUT transporters and are phosphorylated by hexokinase to G6P and M6P, respectively. M6P is either isomerized by MPI to F6P to feed glycolysis/PPP or converted by PMM1/2 to M1P and onward to GDP-mannose for protein *N*-glycosylation, *O*-mannosylation, and glycosylphosphatidylinositol (GPI) anchor biosynthesis.

The dependency of the glycolytic pathway in the bone physiological environment due to low oxygen conditions has been proven. On the other hand, the impact of the monosaccharides metabolic pathway, particularly in the mannose metabolic pathway, on the tumor progression of osteosarcoma remains unclear. First, our attention is on the oncogenic feature of the mannose metabolic pathway in the patient with osteosarcoma. To gain the potential genetic signature of osteosarcoma, we analyzed the cancer genome of osteosarcoma patients using public resources. Of note, glucose and mannose share the GLUT transporter for incorporation into cells from the extracellular environment (Figure 1). In addition, glucose and mannose are phosphorylated by an identical kinase, hexokinase (HK). Thus, glucose and mannose are commonly metabolized at the initial step of the cellular processes. Therefore, we conducted our analysis focusing on both glycolysis and mannose metabolism.

We reanalyzed a total of 18 osteosarcoma patient data sets. Compared to paired normal tissue, only *GPI* mRNA expression was statistically upregulated in osteosarcoma of patients, whereas genes involved in glycolysis and mannose metabolism tended to increase the expression in osteosarcoma tumor tissue (Table 1).

**Figure 2:**
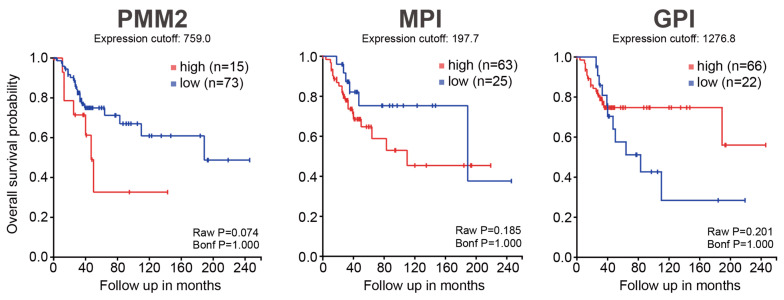
Prognostic impact of glycolysis and mannose metabolism-related gene clusters in osteosarcoma patients. Kaplan–Meier overall-survival plots (GSE42352, *n* = 88) were generated in the R2 Genomics Analysis Platform using scan-mode expression cutoffs compared high vs. low cluster expression. As the trends of the results are noted across the group, there are no statistically significant differences in this cohort.

These results are as expected and consistent with previous reports [14]. Therefore, we decided to verify the relationship between gene expression and patient prognosis using different patient data. A total of 88 osteosarcoma patient data were analyzed using the R2 Genomics Analysis Platform (Figure 2). In this cohort of 88 patients, we did not observe a statistically significant association between the glycolysis or mannose metabolism signatures and overall survival. The glycolysis signature showed a non-significant trend toward shorter survival, whereas *PMM2* and *MPI* showed non-significant trends in the opposite direction. Given scan mode thresholding and the absence of covariate adjustment, we treat these patterns as descriptive and do not infer a prognostic or causal role. Crucially, because these analyses are underpowered and exploratory, no clinical recommendations or prognostic conclusions should be drawn from them. We frame these findings strictly as observations that prompted our in vitro hypothesis, rather than as clinical evidence.

### 3.2. The Impact of Mannose Supplementation on Osteosarcoma Cell Lines

Although mannose supplementation inhibits the proliferation of osteosarcoma cells and dramatically enhances the effects of existing anticancer drugs, the details of its effects and molecular mechanisms remain incompletely defined [11,12]. To identify the action point of mannose supplementation in osteosarcoma, we prepared two types of osteosarcoma cell lines, U-2 OS and MG-63. U-2 OS and MG-63 cell lines can indeed be models representative of a relatively early-to-intermediate stage within the malignant progression spectrum of osteosarcoma, carrying relatively fewer genomic abnormalities.

**Figure 3:**
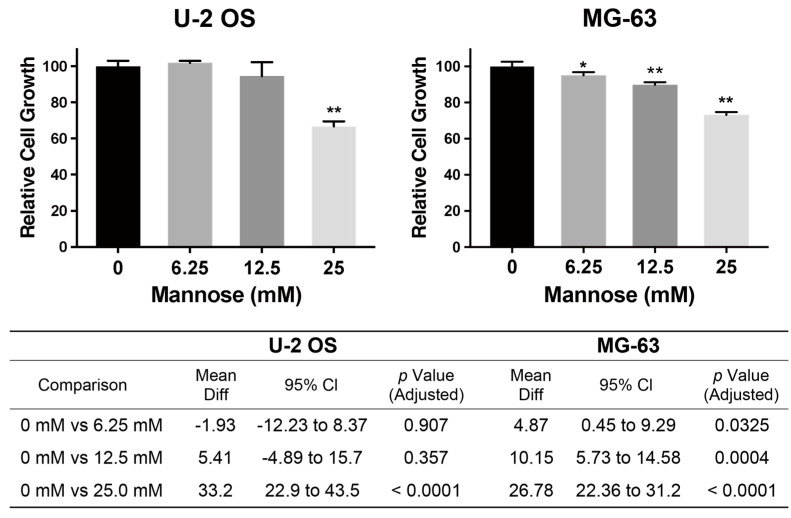
The impact of mannose supplementation on cell growth of osteosarcoma cell lines. After 72 h of D-mannose supplementation (0–25 mM), the relative cell growth of U-2 OS and MG-63 is shown compared to 0 mM (*n* = 4 independent experiments). A significant reduction in cell growth was observed at 25 mM in both cell lines. In MG-63 cells, reduction in cell growth was detected starting from 6.25 mM in a dose-dependent manner. Data are presented as mean ± SD, along with the statistical results assessed by two-way ANOVA followed by Dunnett’s multiple comparison test. * *p* < 0.05, ** *p* < 0.01.

In this study, we position mannose as a mechanistic probe to interrogate HK-M6P-MPI/PMM flux in osteosarcoma cells, following the representative study using mannose supplementation to determine the optimal mannose concentration [11]. Mannose supplementation inhibited the proliferation of osteosarcoma U-2 OS cells, as shown in previous reports, at a concentration of 25 mM [11]. In MG-63 cells, a dose-dependent inhibition of proliferation was observed even in a low concentration of 6.25 mM mannose (Figure 3). Thus, MG-63 cells have higher sensitivity to mannose than U-2 OS.

### 3.3. The Differences in Gene Expression Between U-2 OS and MG-63 Cells

**Figure 4:**
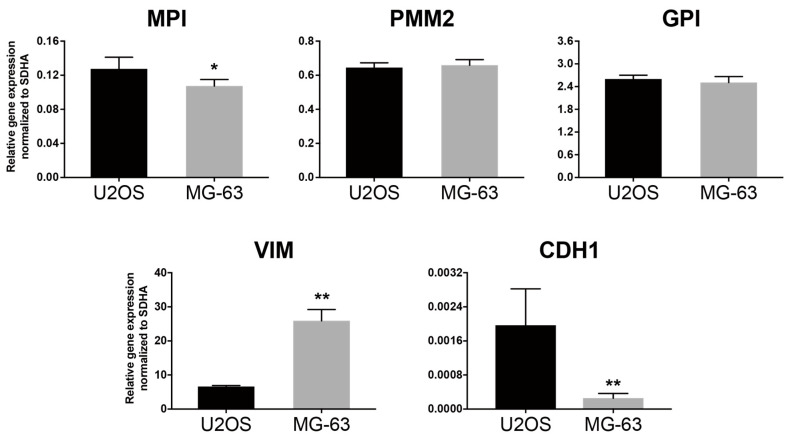
Differential expression of MPI and EMT-related genes in osteosarcoma cell lines. Total RNAs were collected under normal proliferation conditions. qPCR analysis shows significantly lower *MPI* expression and altered EMT-associated gene expression in MG-63 compared to U-2 OS, consistent with their distinct metastatic phenotypes. Data are presented as mean ± SD (*n* = 3) along with the results of unpaired *t*-test. * *p* < 0.05, ** *p* < 0.01.

The differences in mannose sensitivity among U-2 OS and MG-63 may shed light on the mechanism of action point of mannose supplementation in cancer. Therefore, we decided to analyze the differences between U-2 OS and MG-63 based on gene expression. Analysis of the expression of glycolysis and mannose metabolism-related gene clusters revealed that only *MPI* gene expression was significantly lower in MG-63 cells than in U-2 OS cells (Figure 4).

Sensitivity to mannose supplementation depends on MPI expression level, as reported by other groups [11,12,13]. Therefore, it is suggested that the decrease in MPI expression in MG-63 cells is correlated with more potent growth inhibition than in U-2 OS cells. On the other hand, it was found that there was a remarkable difference in the expression levels of EMT-related genes associated with cancer metastasis between U-2 OS and MG-63 cells. These results suggest that the effect of mannose supplementation is strong on highly metastatic cancer cells.

### 3.4. The Effect of Mannose Supplementation on Motility-Associated Phenotypes in MG-63 Cells

**Figure 5:**
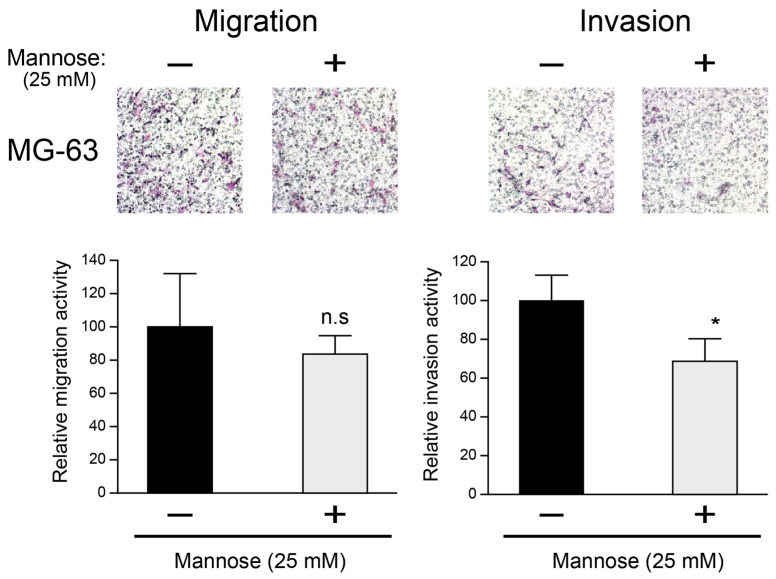
Effect of mannose supplementation on migration and invasion in MG-63 cells. Transwell assays conducted with 25 mM mannose for 48 h demonstrate that mannose supplementation reduces the invasive ability of MG-63 cells, whereas the reduction in migration is not statistically significant. Representative images are shown. Data are presented as relative percentages to control (*n* = 3), along with the results of a paired *t*-test. * *p* < 0.05, n.s: Not significant.

After treatment with 25 mM mannose, we examined the ability of migration and invasion of MG-63 cells. The mannose supplementation moderately decreased the migration ability of MG-63, while potent inhibition of invasive ability was observed (Figure 5). Under the same 25 mM mannose condition, the cell growth at 72 h was decreased but did not suggest overt cytotoxicity by microscopy, as we did not observe widespread cell detachment or gross loss of adherent cells. Therefore, the reduced migration and invasion readouts are unlikely to be explained solely by acute cell death, although contributions from altered proliferation and metabolic state cannot be excluded.

These results indicate that mannose supplementation is associated with reduced proliferative capacity and with decreased migration and invasion in MG-63 cells. However, because cell viability was not directly measured under the migration and invasion assay conditions, we cannot exclude the contribution of reduced cell number to these readouts. Therefore, the observed reduction in migrated and invaded cell numbers may reflect a composite effect of altered motility, proliferation, and/or metabolic state under these assay conditions.

### 3.5. Generation of MPI-Knockdown Cells in MG-63 Cells

**Figure 6:**
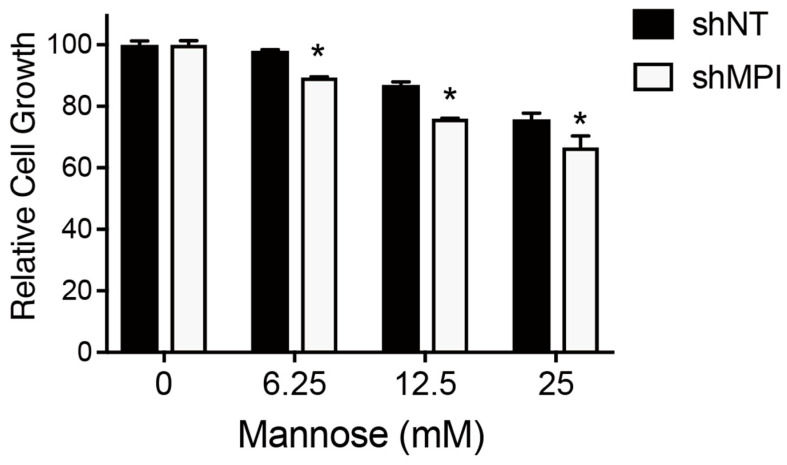
Knockdown of MPI enhances the inhibitory effect of mannose supplementation on cell growth in MG-63 cells. MG-63 cells (shNT and shMPI) were treated with mannose (0–25 mM) for 72 h. Mannose supplementation inhibited cell proliferation in a dose-dependent manner. The effect of proliferation inhibition was greater in MPI-knockdown cells compared to control cells (shNT). Data are presented as mean ± SD (*n* = 3), along with the results of statistical analysis by two-way ANOVA followed by Dunnett multiple comparison test. * *p* < 0.05.

To gain insight into the action point of mannose supplementation in MG-63 cells, we undertook the knockdown approach for MG-63 cells. To date, it is known that the *MPI* gene plays a key role in the effects of mannose supplementation on cancer cells. Therefore, we attempted to generate the *MPI*-knockdown cells derived from MG-63 cells. Five short hairpin RNA (shRNA) expression vectors predesigned for *MPI* were prepared. First, we validate the knockdown effect of each shRNA-expressing vector in a cell with high-transfection efficiency by transient transfection. In this experiment, we confirmed the enough effect of MPI knockdown (Figure S1). Based on these results, we tried to generate a series of the MPI-knockdown cells in MG-63 cells. After transfection of the vector, cells were cultured in the presence of puromycin to select shRNA-introduced MG-63 cells. A non-targeting shRNA (shNT) was used as a control. As a result, only two kinds of cells with shRNA expression vectors specific to the *MPI* gene survived, and then the expression levels of the *MPI* gene were examined. Unfortunately, one shRNA (shMPI#4) only showed a 40% reduction in *MPI* expression (Figure S2). We therefore employed the shMPI#4-expressing cells to investigate the effect of *MPI* knockdown on cell growth. The inhibitory effect of cell growth by mannose supplementation appeared more pronounced in *MPI* knockdown cells (Figure 6). These results are consistent with those previously reported in U-2 OS cells.

### 3.6. Effect of Mannose Supplementation on Motility-Associated Phenotypes in MPI-Knockdown Cells

**Figure 7:**
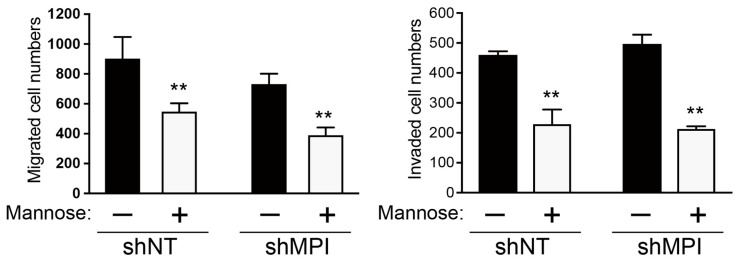
Knockdown of *MPI* enhances the inhibitory effect of mannose supplementation on metastatic properties of MG-63 cells. MG-63 cells (shNT and shMPI) were treated with 25 mM mannose for 48 h. Mannose supplementation inhibited cell migration and invasion. *MPI*-knockdown MG-63 cells show trends consistent with mannose sensitivity, displaying modest reductions in migration and invasion compared to control cells. Data are presented as mean ± SD (*n* = 3), along with the results of paired *t*-test. ** *p* < 0.01.

Next, we examined whether the *MPI* knockdown modules the reduction in motility-associated phenotypes induced by mannose supplementation. Interestingly, the inhibitory effect of migration and invasion activity by mannose supplementation was also observed in both control (shNT) and *MPI*-knockdown cells (Figure 7). These results indicate that mannose supplementation reduces migration and invasion activity in a manner consistent with MPI-modulated sensitivity, although the partial knockdown precludes a definitive demonstration of additive efficacy.

### 3.7. Effect of MPI Knockdown and Mannose Supplementation on the Expression of Metastasis-Related Genes

**Figure 8:**
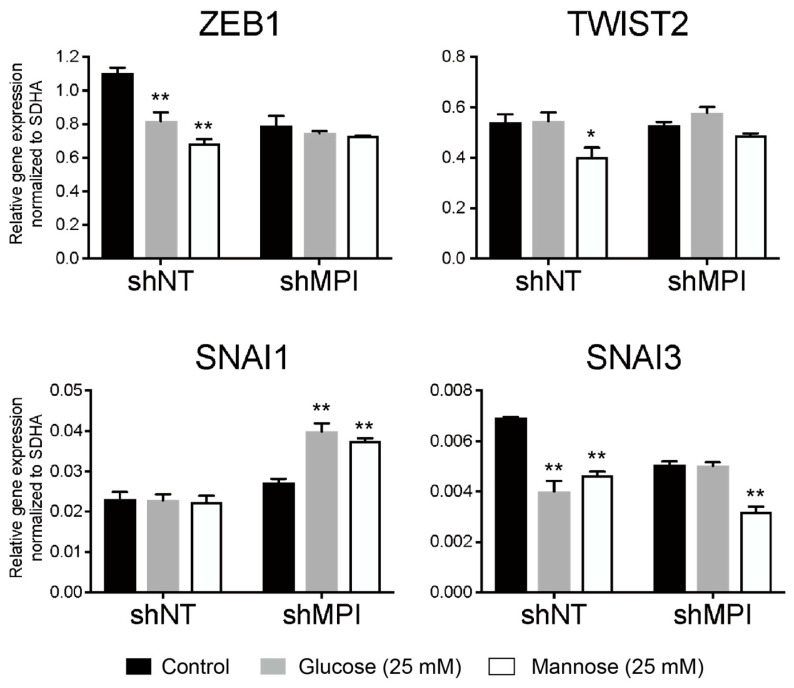
Alteration of gene expression of MG-63 cells in response to glucose and mannose supplementation. MG-63 cells were treated with 25 mM glucose or 25 mM mannose for 48 h. qPCR analysis shows that mannose supplementation downregulates metastasis-associated genes, including EMT markers, indicating a transcriptional basis for reduced malignancy. Data are presented as mean ± SD (*n* = 3), along with the results of statistical analysis by one-way ANOVA followed by Dunnett multiple comparison test. * *p* < 0.05, ** *p* < 0.01.

To elucidate the mechanism by which *MPI* knockdown and mannose supplementation suppress cancer motility-associated phenotypes, we evaluate the expression dynamics of genes related to cancer metastasis. As MG-63 cells showed expression of EMT markers (Figure 4), we examined the expression of metastasis-related gene clusters associated with EMT (Figure 8).

In shNT cells of MG-63, ZEB1 gene expression decreased upon supplementation of either glucose or mannose. However, these decreases reached the steady-state level of shMPI cells. On the other hand, in shMPI cells, the supplementation of glucose and mannose had no effect on ZEB1 gene expression. In TWIST2 gene expression, shNT cells selectively suppressed expression upon mannose supplementation. However, this selective suppression was not observed in shMPI cells. Unexpectedly, SNAI1 gene expression was induced by the addition of glucose or mannose in shMPI cells, but this effect was not observed in shNT cells. Most interestingly, SNAI3 gene expression decreased in both shNT and shMPI cells upon mannose supplementation. Taken together, mannose supplementation seems to reduce cell motility and invasion by regulating the expression of multiple metastasis-related genes rather than by suppressing the expression of specific genes. Furthermore, mannose supplementation affected these models by modulating glycolysis-associated outputs that were sensitive to MPI knockdown, while also reducing proliferation-associated and motility-associated readouts through mechanisms that remain unresolved.

## 4. Discussion

Mannose supplementation has gained attention as an attractive therapeutic strategy, with anti-tumor effects confirmed across several types of cancers in vitro and in vivo [15]. However, the molecular mechanism of the lethal effect on cancer cells due to mannose supplementation remains unclear. Regarding mannose metabolism and lethality, it is known as a classic honeybee syndrome, and the only clue is its association with the expression of MPI, a mannose metabolism enzyme, and similar effects are observed in cancer cells. This study provides an additional effect of mannose supplementation on cancer cells. Of note, we found that mannose supplementation leads not only to the reduction in cancer cell proliferation but also to a decrease in the expression of genes involved in cancer metastasis, thereby reducing motility and invasiveness. While we did not quantify endpoint viability under the transwell conditions, the mannose exposures used here did not produce gross cytotoxic morphology in parallel 72 h cultures, suggesting that the motility-associated reductions are not attributable only to acute cell death.

Osteosarcoma is known to exhibit a pronounced increase in glycolysis, a characteristic of cancer cells, due to the tissue-specific hypoxic environment [5]. Therefore, the mannose metabolic pathway, which shares the transporter with the glycolytic pathway, might increase the risk of cancer to adapt to the environmental and cancer-specific characteristics, but it remains unclear. The difficulty in osteosarcoma research lies in the limited availability of patient data. In this study, no statistically significant differences were found, although specific trends were observed in the analysis of patient prognosis data. This might be likely due to the small number of patients in the dataset. Availability and collectability of the data from osteosarcoma patients may overcome the bottleneck of osteosarcoma research based on patient data in the future.

By utilizing osteosarcoma cell lines, we observed that mannose supplementation suppresses metastasis-associated transcripts and reduces migration and invasion readouts in vitro. Because we did not directly quantify mannose flux, MPI activity, or pathway-level protein effectors, these findings should be interpreted as hypothesis-generating rather than as evidence for an established MPI-independent mechanism.

It should be noted that the millimolar concentrations of mannose used in this study (up to 25 mM) exceed typical physiological plasma levels. We employed these concentrations strictly as a mechanistic probe to reveal metabolic vulnerabilities and flux dependencies in osteosarcoma cells in vitro, rather than to simulate clinical pharmacokinetics. These findings identify mannose metabolism as a targetable pathway, suggesting that future therapeutic strategies might involve enzymatic inhibition or targeted delivery rather than simple dietary supplementation.

Here, we use the term ‘collateral effect’ to denote the co-occurrence of altered motility-associated readouts and EMT-associated transcripts under mannose exposure; it does not imply a defined mechanism. Elucidating the molecular mechanism of the collateral effect of mannose supplementation may lead not only to a better understanding of the importance of mannose supplementation but also to the development of future mechanistic studies. *Zeb1* and *Twist2* genes analyzed in this study are controlled by pathways that are important for cancer metastasis, such as TGF-β and Wnt signaling [16]. Since the expression dynamics of Zeb1 and Twist2 differ depending on mannose supplementation, it is possible that these gene expressions would be regulated by different pathways or other mechanisms. In *MPI* knockout cells, the reason why only *SNAI1* showed increased expression upon either glucose or mannose supplementation is unclear, but it is possible that the balance between glycolytic signaling and other intracellular signaling was affected. Unfortunately, despite the shRNA vector itself being effective, only one out of five shRNAs used in this study’s knockdown approach was successful. Thus, we fail to generate a stable, well-knockdown of the MPI gene in MG-63 osteosarcoma cells. The most likely reason is that effective *MPI* knockdown is highly likely to induce ER stress and growth arrest/retardation of cells. It matches the honeybee syndrome, in which the MPI- deficient cells die early, and partially silenced survivors repopulate [17]. Consistent with this, we generated an *MPI*-knockdown MG-63 cell line with partial knockdown. Additionally, no group has shown the generation of MPI-knockdown or knockout in MG-63 or U-2 OS, whereas several studies have achieved depletion in other cell types, including mouse melanoma B16-F1 and mouse lung carcinoma LLC [11]. Therefore, effective knockdown in osteosarcoma cells might induce toxicity or lethality in a context-dependent manner. On the other hand, partial knockdown allows us to observe the metabolic ‘collateral’ effects under viable conditions. However, we explicitly acknowledge that this partial suppression limits our ability to strictly decouple MPI-dependent from MPI-independent effects. Therefore, we proceeded with our research, viewing this limited knockdown as valuable in its own right, without fixating on knockdown efficiency. Based on these points, validation using other shRNAs will be addressed in future studies. Furthermore, this study only verified gene expression using qPCR, and protein expression should also have been examined. But due to limitations in the availability of antibodies, this was not examined in this study.

Our data suggest that mannose exposure can modulate EMT-associated transcriptional programs and motility-associated phenotypes in vitro, including reduced *ZEB1* and *TWIST2* expression. Related pathway-level effects have been reported in other tumor contexts, including signaling modules that regulate β-catenin and EMT [18], but we did not assay these pathways in osteosarcoma cells and therefore discuss them as candidate mechanisms rather than established drivers.

In other reports, mannose supplementation has also been shown to alter the UDP-GlcNAc pool and to modify OGT-dependent O-GlcNAcylation, thereby diminishing the transcriptional activity of β-catenin and YAP [19,20]; these pathways were not measured here. Because the YAP–CARM1 pathway is implicated in doxorubicin resistance in osteosarcoma [21], the enhanced mannose sensitivity observed in our model could be consistent with YAP suppression driven by altered O-GlcNAc modification. Critically, distinguishing MPI-dependent from MPI-independent contributions in osteosarcoma will require targeted mechanistic experiments. These should include measurement of MPI protein abundance and enzymatic activity, quantitative assessment of UPR activation markers, quantification of global O-GlcNAc levels, and protein-level analysis of YAP and β-catenin abundance and subcellular localization under mannose supplementation.

From a clinical standpoint, a recent integrated transcriptome study in osteosarcoma reported that a glycolytic gene signature, including *HK2*, *MPI*, and *GPI*, correlates with poor prognosis [22]. We focused on specific genes at the intersection of glycolysis and mannose metabolism. However, broader pathway analysis like GSEA will be essential in future studies to fully map the transcriptomic shifts.

Mannose supplementation might exert an impact to osteosarcoma as a novel “collateral effect”, suppressing tumor growth and motility-associated phenotypes. These effects likely involve a composite of metabolic factors modulated by MPI expression, rather than a singular linear mechanism. In our in vitro assays, mannose supplementation decreased the CCK-8 growth readout and reduced transwell migration and invasion readouts, accompanied by decreased expression of EMT-associated transcripts. The extent to which these phenotypes are mediated by MPI-linked mannose metabolism versus additional pathways remains to be determined, and candidate mechanisms reported in other systems—including UPR activation, altered O-GlcNAc signaling, and downstream regulators such as YAP or β-catenin—were not directly measured in this study and should be tested in future work.

While our current findings provide in vitro observations consistent with mannose metabolism in osteosarcoma, the absence of in vivo validation remains a significant limitation of this study. Future research using orthotopic and experimental lung and bone metastasis mouse models via tail vein injection will be required to evaluate and confirm the therapeutic efficacy of mannose within the complex bone microenvironment. Future work should define which phenotypes are driven by MPI-linked mannose metabolism versus additional pathways using in vivo models and mechanistic rescue experiments. Key experiments include MPI protein and activity measurements, protein-level quantification of UPR activation, assessment of global O-GlcNAcylation, and YAP and β-catenin signaling analyses, ideally integrated with isotope-tracing-based flux measurements.

## 5. Conclusions

Our findings identify mannose metabolism as a tractable vulnerability in osteosarcoma, demonstrating that supplementation exerts a collateral suppressive effect on both proliferation and motility-associated phenotypes through MPI-dependent modulation of epithelial-to-mesenchymal transition drivers.

## Figures and Tables

**Table 1:** Differential expression between the pair of normal and tumor in osteosarcoma patients.

Gene	Normalized Counts (Means)		log2FC	Stat ^1^	*p* Value ^2^	*p* Value (*adj*) ^3^
	Normal	Tumor				
*HK2*	192.8	230.4	0.256	0.957	0.339	0.556
*MPI*	36.5	46.0	0.315	2.110	0.035	0.144
*GPI*	322.6	417.7	0.643	2.743	0.006	0.048 *
*PMM1*	28.2	37.0	0.375	1.879	0.060	0.200
*PMM2*	49.2	62.1	0.324	1.817	0.069	0.219
*GMPPB*	11.8	16.8	0.560	2.387	0.017	0.091

^1^ Wald statistic, ^2^ Wald test *p*-value, and ^3^ BH adjusted *p*-value are calculated by DESeq2. * *p* < 0.05.

## Data Availability

The original contributions presented in this study are included in the article/Supplementary Materials. Further inquiries can be directed to the corresponding author.

## Supplementary Materials

The following supporting information can be downloaded at https://www.mdpi.com/article/10.3390/biology15020127/s1, Table S1: DNA sequences of the primers for qPCR., Figure S1: Validation of the shRNA vectors to MPI-knockdown by transient transfection. Figure S2: Expression levels of the MPI gene in a stable knockdown MG-63 cell lines.

## Abbreviations

The following abbreviations are used in this manuscript:

GDP	Guanosine diphosphate
PET	Positron emission tomography
ATP	Adenosine triphosphate
dNTP	Deoxyribonucleotides
qPCR	Quantitative polymerase chain reaction
FCS	Fetal calf serum
ANOVA	Analysis of variance

## References

[1] Mirabello L., Troisi R.J., Savage S.A. (2009). Osteosarcoma incidence and survival rates from 1973 to 2004: Data from the Surveillance, Epidemiology, and End Results Program. Cancer.

[2] Bielack S.S., Kempf-Bielack B., Delling G., Exner G.U., Flege S., Helmke K., Kotz R., Salzer-Kuntschik M., Werner M., Winkelmann W. (2002). Prognostic factors in high-grade osteosarcoma of the extremities or trunk: An analysis of 1,702 patients treated on neoadjuvant cooperative osteosarcoma study group protocols. J. Clin. Oncol..

[3] Warburg O. (1956). On the origin of cancer cells. Science.

[4] Heiden M.G., Cantley L.C., Thompson C.B. (2009). Understanding the Warburg effect: The metabolic requirements of cell proliferation. Science.

[5] Kansara M., Teng M.W., Smyth M.J., Thomas D.M. (2014). Translational biology of osteosarcoma. Nat. Rev. Cancer.

[6] Hamada K., Tomita Y., Inoue A., Fujimoto T., Hashimoto N., Myoui A., Yoshikawa H., Hatazawa J. (2009). Evaluation of chemotherapy response in osteosarcoma with FDG-PET. Ann. Nucl. Med..

[7] Zhang C., Chen H., Xu Y., Li X., Wang L., Sun J. (2019). Effectiveness of ^18^F-FDG PET/CT in the diagnosis and staging of osteosarcoma: A systematic review and meta-analysis. BMC Cancer.

[8] Kaelin W.G., Ratcliffe P.J. (2008). Oxygen sensing by metazoans: The central role of the HIF hydroxylase pathway. Mol. Cell.

[9] Vousden K.H., Ryan K.M. (2009). p53 and metabolism. Nat. Rev. Cancer.

[10] Sharma V., Ichikawa M., Freeze H.H. (2014). Mannose metabolism: More than meets the eye. Biochem. Biophys. Res. Commun..

[11] Gonzalez P.S., O’Prey J., Cardaci S., Barthet V.J., Sakamaki J.I., Beaumatin F., Roseweir A., Gay D.M., Mackay G., Malviya G. (2018). Mannose impairs tumour growth and enhances chemotherapy. Nature.

[12] Harada Y. (2025). Manipulating mannose metabolism as a potential anticancer strategy. FEBS J..

[13] Harada Y., Mizote Y., Suzuki T., Hirayama A., Ikeda S., Nishida M., Hiratsuka T., Ueda A., Imagawa Y., Maeda K. (2023). Metabolic clogging of mannose triggers dNTP loss and genomic instability in human cancer cells. eLife.

[14] Feng Z., Ou Y., Hao L. (2022). The roles of glycolysis in osteosarcoma. Front. Pharmacol..

[15] Zhou X., Song Y., Wang Z., Fu L., Xu L., Feng X., Zhang Z., Yuan K. (2025). Dietary sugar intervention: A promising approach for cancer therapy. Biochim. Biophys. Acta Rev. Cancer.

[16] Sols A., Cadenas E., Alvarado F. (1960). Enzymatic basis of mannose toxicity in hone bees. Science.

[17] Lebeaupin C., Yong J., Kaufman R.J. (2020). The Impact of the ER Unfolded Protein Response on Cancer Initiation and Progression: Therapeutic Implications. Adv. Exp. Med. Biol..

[18] Luo Q., Li B., Li G. (2020). Mannose Suppresses the Proliferation and Metastasis of Lung Cancer by Targeting the ERK/GSK-3β/β-Catenin/SNAIL Axis. OncoTargets Ther..

[19] Peng C., Zhu Y., Zhang W., Liao Q., Chen Y., Zhao X., Guo Q., Shen P., Zhen B., Qian X. (2017). Regulation of the Hippo-YAP Pathway by Glucose Sensor O-GlcNAcylation. Mol. Cell.

[20] Olivier-Van Stichelen S., Dehennaut V., Buzy A., Zachayus J.L., Guinez C., Mir A.M., Yazidi-Belkoura E.l.-I., Copin M.C., Boureme D., Loyaux D. (2014). O-GlcNAcylation stabilizes beta-catenin through direct competition with phosphorylation at threonine 41. FASEB J..

[21] Li Z., Lu H., Zhang Y., Lv J., Zhang Y., Xu T., Yang D., Duan Z., Guan Y., Jiang Z. (2024). Blocking CXCR4-CARM1-YAP axis overcomes osteosarcoma doxorubicin resistance by suppressing aerobic glycolysis. Cancer Sci..

[22] Zhu N., Hou J., Zhang Y., Yang N., Ding K., Chang C., Liu Y., Gu H., Chen B., Wei X. (2025). A prognostic glycolysis-related gene signature in osteosarcoma: Implications for metabolic programming, immune microenvironment, and drug response. PeerJ.

