# Supplementary material for: Collateral Impact of Mannose Supplementation on Metastatic Properties in Osteosarcoma Cell Models
Source: Biology (Basel). 2026 Jan 11;15(2):127. doi: 10.3390/biology15020127 (PMC12837248; doi:10.3390/biology15020127)
Supplement: Supplementary file 1 [file biology-15-00127-s001.zip › Figure S1.pdf]

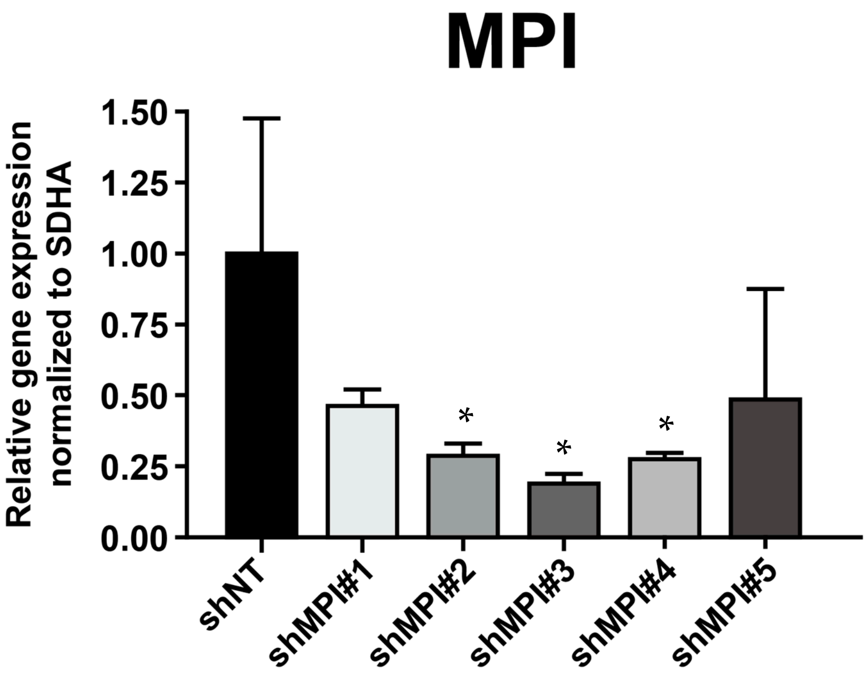

Figure S1. Validation of the shRNA vectors to MPI-knockdown by transient transfection. Knockdown efficiencies of five shRNA vectors were examined by transient transfection. RNAs were isolated, and then qPCR was performed. The levels of MPI expression were normalized to SDHA. Data are presented as mean ± SD, along with multiple-comparison statistics. * $P < 0.05$
