# Supplementary material for: Collateral Impact of Mannose Supplementation on Metastatic Properties in Osteosarcoma Cell Models
Source: Biology (Basel). 2026 Jan 11;15(2):127. doi: 10.3390/biology15020127 (PMC12837248; doi:10.3390/biology15020127)
Supplement: Supplementary file 1 [file biology-15-00127-s001.zip › Figure S2.pdf]

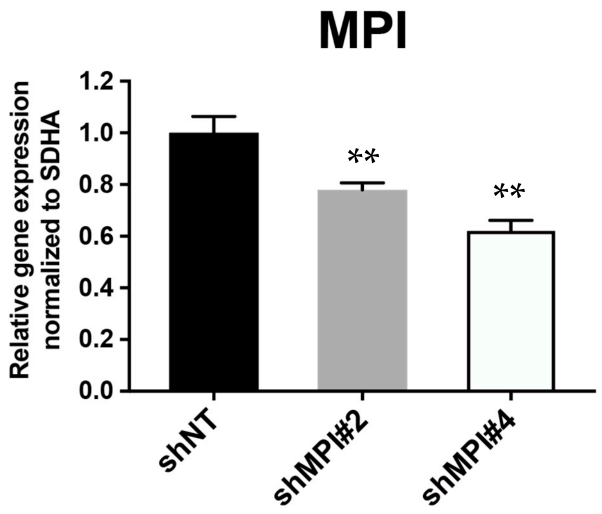

Figure S2 Expression levels of the MPI gene in a stable knockdown MG-63 cell lines. RNAs were isolated from each cell lines, and then qPCR was performed. The levels of MPI expression were normalized to SDHA. Data are presented as mean ± SD, along with multiple-comparison statistics. ** $P < 0.05$
