# Supplementary material for: Collateral Impact of Mannose Supplementation on Metastatic Properties in Osteosarcoma Cell Models
Source: Biology (Basel). 2026 Jan 11;15(2):127. doi: 10.3390/biology15020127 (PMC12837248; doi:10.3390/biology15020127)
Supplement: Supplementary file 1 [file biology-15-00127-s001.zip › Table S1.pdf]

Table S1. DNA sequences of the primers for qPCR.

Primer name	DNA sequences (5’ -> 3’)
SDHA Fw	TGGGAACAAGAGGGCATCTG
SDHA Rv	CCACCACTGCATCAAATTCATG
MPI Fw	GCATCTCACAGAAGACCCTAAG
MPI Rv	AGAGCACTTTGAAGAGGAAGG
PMM2 Fw	CTTCGACGTGGATGGGACC
PMM2 Rv	CGCCTACCACTCCGATTTTG
GPI Fw	AGGCTGCTGCCACATAAGGT
GPI Rv	AGCGTCGTGAGAGGTCACTTG
VIM Fw	CGGGAGAAATTGCAGGAGGA
VIM Rv	AAGGTCAAGACGTGCCAGAG
CDH1 Fw	ATGAGTGTCCCCCGGTATCT
CDH1 Rv	GGTCAGTATCAGCCGCTTTC
ZEB1 Fw	CATTTTTCCTGAGGCACCTG
ZEB1 Rv	TGAAAATGCATCTGGTGTTCC
TWIST2 Fw	GCGCCAGGGCTGTCC
TWIST2 Rv	GGCCTCCTGAGGTTGTTCAG
SNAI1 Fw	TCGGAAGCCTAACTACAGCGA
SNAI1 Rv	AGATGAGCATTGGCAGCGAG
SNAI3 Fw	AAGATGCACATCCGCACTCA
SNAI3 Rv	ATAGGGCTTCTCCCCTGTGT
